# Up-regulation of miR-381 inhibits NAD+ salvage pathway and promotes apoptosis in breast cancer cells

**DOI:** 10.17179/excli2019-1431

**Published:** 2019-08-27

**Authors:** Zahra Bolandghamat Pour, Mitra Nourbakhsh, Kazem Mousavizadeh, Zahra Madjd, Seyedeh Sara Ghorbanhosseini, Zohreh Abdolvahabi, Zahra Hesari, Samira Ezzati Mobaser

**Affiliations:** 1 Department of Molecular Medicine, Faculty of Advanced Technologies in Medicine, Iran University of Medical Sciences, Tehran, Iran; 2 Department of Biochemistry, School of Medicine, Iran University of Medical Sciences, Tehran, Iran; 3 Cellular and Molecular Research Center, Faculty of Medicine, Iran University of Medical Sciences, Tehran, Iran; 4 Oncopathology Research Center, Iran University of Medical Sciences, Tehran, Iran; 5 Department of Biochemistry and Genetics, Cellular and Molecular Research Center, Qazvin University of Medical Sciences, Qazvin, Iran; 6 Laboratory Sciences Research Center, Golestan University of Medical Sciences, Gorgan, Iran; 7 Department of Laboratory Sciences, Faculty of Paramedicine, Golestan University of Medical Sciences, Gorgan, Iran

**Keywords:** nicotinamide phosphoribosyltransferase, nicotinamide adenine dinucleotide, miR-381, apoptosis, breast cancer, tumor suppression

## Abstract

Nicotinamide phosphoribosyltransferase (NAMPT), a rate-limiting enzyme involved in nicotinamide adenine dinucleotide (NAD) salvage pathway, is overexpressed in many human malignancies such as breast cancer. This enzyme plays a critical role in survival and growth of cancer cells. MicroRNAs (miRNAs) are among the most important regulators of gene expression, and serve as potential targets for diagnosis, prognosis, and therapy of breast cancer. Therefore, the aim of this study was to assess the effect of NAMPT inhibition by miR-381 on breast cancer cell survival. MCF-7 and MDA-MB-231 cancer cell lines were transfected with miR-381 mimic, inhibitor, and their corresponding negative controls (NCs). Subsequently, the level of NAMPT and NAD was assessed using real-time PCR, immuno-blotting, and enzymatic methods, respectively. In order to evaluate apoptosis, cells were labelled with Annexin V-FITC and propidium iodide and analyzed by flow cytometry. Bioinformatics analysis was performed to recognize whether NAMPT 3′-untranslated region (UTR) is a direct target of miR-381 and the results were authenticated by the luciferase reporter assay using a vector containing the 3′-UTR sequence of NAMPT. Our results revealed that the 3′-UTR of NAMPT was a direct target of miR-381 and its up-regulation decreased NAMPT gene and protein expression, leading to a notable reduction in intracellular NAD and subsequently cell survival and induction of apoptosis. It can be concluded that miR-381 has a vital role in tumor suppression by down-regulation of NAMPT, and it can be a promising candidate for breast cancer therapy.

## Introduction

Cancer represents one of the main life-threatening diseases all over the world (Kyu-Won Jung and Lee, 2018[15]). It has been the cause of 9.6 million deaths in 2018, approximately one in six individuals worldwide (http://www.who.int/news-room/fact-sheets/detail/cancer). There are over 200 different types of cancers and the most common types include lung, breast, colorectal, prostate, skin and stomach cancer (http://www.who.int/news-room/fact-sheets/detail/cancer; Jainish and Prittesh, 2017[13]). Despite the advances in screening and early detection of breast cancer, it is the most commonly occurring cancer in women and is the second leading cause of cancer-related deaths after lung cancer (Zhou et al., 2013[36]). According to the American Cancer Society estimations, in 2017 about 252,710 new cases of invasive breast cancer were diagnosed in women and about 40,610 women died from this cancer (Hashemi et al., 2018[10]; Baldassari et al., 2018[5]). Therefore, there is an urgent need for discovering new approaches for the treatment of breast cancer. Identification of the related molecular changes can be helpful in creating novel diagnostic and therapeutic strategies for this malignancy (Zhou et al., 2013[36]). Nicotinamide adenine dinucleotide (NAD) is a necessary component for living organisms and plays a significant role in biological processes such as DNA repair, gene transcription regulation, oncogenic signal transduction, cell cycle progression, genomic integrity, apoptosis, and metabolism (Xu et al., 2017[31]; Vora et al., 2016[25]). It is also a critical coenzyme for redox reactions in cancer cell metabolic pathways and promotes tumor survival and progression by regulating multiple cellular functions (Vora et al., 2016[25]). Biochemically, there are three main pathways for the formation of NAD in eukaryotic cells including catabolism of tryptophan and two alternative salvage pathways (Sampath et al., 2015[22]). The primary salvage pathway in producing NAD is catalyzed by nicotinamide phosphoribosyltransferase (NAMPT) enzyme (Sampath et al., 2015[22]). NAMPT, also known as pre-B cell colony-enhancing factor 1 or visfatin, is a rate-limiting enzyme in the salvage pathway of (NAD) biosynthesis (Zhou et al., 2014[38]; Dalamaga et al., 2018[9]). This enzyme is up-regulated in many different human malignancies including breast*, *prostate, colon, thyroid, gastric and obesity-associated cancers (Dalamaga et al., 2018[9]; Vora et al., 2016[25]; Chen et al., 2013[8]). Therefore, NAMPT can be an ideal cancer marker and the pharmacologic agents or molecular mechanisms that decrease its level are suitable candidates for anti-cancer treatments (Dalamaga et al., 2018[9]). Small non-coding RNAs (18-22 nucleotides in length), known as microRNAs (miRNAs), are the key regulators of gene expression and modulate up to one-third of all genes. The function of miRNAs is exerted via their interaction with 3′ untranslated regions (3′-UTR) of their target genes (Zhou et al., 2013[36]; Setijono et al., 2018[23]). miRNAs play an important role in the regulation of various cellular functions such as cell growth, proliferation, differentiation, apoptosis, and metabolism. Therefore, their aberrant expression is linked to many malignancies (Zhou et al., 2013[36]; Zedan et al., 2018[33]; Li et al., 2018[16]). They appeared to be laboratory biomarkers and may represent novel targets for cancer therapy (Setijono et al., 2018[23]; Komatsu et al., 2018[14]). In the present study, we performed a bioinformatics analysis and found that there is a binding site for miR-381 in the 3′-UTR of NAMPT mRNA. Accordingly, we aimed to investigate whether miR-381 up-regulation was associated with a significant decrease in NAD levels and, in turn, the suppression of breast cancer cells via targeting of the NAMPT 3′-UTR. 

## Materials and Methods

### Cell culture 

HEK-293T, MCF-7, MCF-10A, and MDA-MB-231 cell lines were obtained from the Cell Bank of the Iranian Biological Resource Centre (Tehran, Iran). MCF-7 and MDA-MB-231 were maintained in Dulbecco's modified Eagle medium (DMEM, Biosera, France) with 10 % fetal bovine serum (FBS) (Invitrogen, UK), penicillin (100 units/ml) and streptomycin (100 μg/ml). MCF-10A cells were cultured in mammary epithelial cell growth medium (MEGM; Lonza/Clonetics, Switzerland) supplemented with 100 ng/ml cholera toxin (CT) (Sigma-Aldrich, Germany), 10 μg/ml insulin (Sigma-Aldrich), 20 ng/ml epithelial growth factor (EGF) (Sigma-Aldrich), 0.5 μg/ml hydrocortisone (HC) (Sigma-Aldrich), 10 % FBS and 1 % penicillin-streptomycin. In the case of HEK-293T, cells were maintained in DMEM/F12 (Biosera, France) medium, supplemented with 1 % penicillin-streptomycin and 10 % FBS. All cell lines were kept in a 5 % CO_2_ humidified incubator at 37 °C.

### Transfection with miR-381 mimic and inhibitor

miR-381 mimic, miR inhibitor and the NCs were purchased from GenePharma (Shanghai, China) and used to respectively increase and decrease the level of miR-381 in MCF-7 and MDA-MB-231 cell lines. The sequences of the employed oligonucleotides are listed in Table 1(Tab. 1). Transfection was achieved using polyethylenimine (PEI, Sigma-Aldrich, Germany). Briefly, cells were seeded into 6-, 12- or 96-well plates and incubated for 24 h. The media was gently aspirated, FBS- and antibiotics-free DMEM was added to each well and incubated for an extra 4 h under the same condition. PEI was mixed with DNA in Opti-MEM (Gibco, UK) with a 1:3 ratio and incubated for 40 min at room temperature (RT) before it was added to the cells. The transfection medium was added to each well and incubated for 4 h. Subsequently, the supernatant was discarded and replaced with fresh complete DMEM and incubated for 48 h prior to further investigations. To determine the transfection efficiencies, cells were transfected with FAM-labeled miRNA and subsequently visualized under fluorescent microscope 8-24 h after transfection. Meanwhile, for quantitative analysis of transfection, the fluorescent images were elaborated using ImageJ 1.51n image processing software (ImageJ, National Institute of Health, Bethesda, Maryland, USA).

### RNA isolation and Real-Time PCR analysis

The expression level of miR-381 and NAMPT mRNA was measured by real-time PCR (RT-PCR). Primarily, total RNA was extracted from cultured cells using miRCURY™ RNA isolation kit (Exiqon, Germany) and its concentration was estimated using NanoDrop spectrophotometer (Nanodrop, Thermo Fisher Scientific, USA). All the procedures were performed according to the manufacturer's instructions. Complementary DNA was synthesized using RevertAid first strand cDNA synthesis kit (Thermo Fisher Scientific, USA) following manufacturer's instructions. In order to synthesize miR-381 cDNA, the 3′-end of miRNA transcripts was polyadenylated by *E. coli *Poly (A) Polymerase (PAP) (New England Biolabs, UK). The tailed miRNAs were reverse transcribed using a hybrid oligo(dT)_12_ primer, wherein 12 residues of dT were linked to a unique adapter sequence. The resulting cDNA was further amplified using a specific forward primer and a universal reverse primer hybridizing to the adapter sequence. To measure miR-381 and NAMPT mRNA levels, RT-PCR was carried out using SYBR Green kit (SYBR Premix Ex Taq II, TaKaRa, Japan) and ABI Detection System (Applied Biosystems, USA). Glyceraldehyde 3-Phosphate Dehydrogenase (GAPDH) and Human U6 small nuclear RNA were used to normalize NAMPT and miR-381 expression levels, respectively. Primer sequences are listed in Supplementary Table 1. The 2^ -ΔΔCT^ method was used to determine the expression levels relative to the internal controls. 

### Cell survival assay 

Cell viability was evaluated with the tetrazolium-based WST-1 cell survival assay kit (Roche Applied Science, Germany) according to the manufacturer's instructions. The MCF-7 and MDA-MB-231 cells were seeded at the density of 5×10^3 ^cells/well in 96-well plates and incubated at 37 °C, with 5 % CO_2_ overnight. Transfection was carried out using miR-381 mimic, miR-381 inhibitor and the relevant NCs as described earlier. Subsequently, 10 µL WST-1 was added to each well and the cells were incubated for 4 h. The optical density (OD) of the formed soluble formazan was measured at 450 nm with 650 nm as the reference wavelength using a plate reader (BioTek Instruments Inc., Winooski, USA). 

### Apoptosis assay 

The effect of miR-381 on cell apoptosis was assessed using Annexin V-FITC apoptosis detection kit (Roche Applied Science, Germany) following the manufacturer's instructions. Briefly, MCF-7 and MDA-MB-231 cells at a density of 3×10^5^ were seeded into a 6-well plate and incubated for 24 h at 37 °C, with 5 % CO_2_. Subsequently, cells were transfected with miR-381 mimic, miR-381 inhibitor and their NCs as described above. After 48 h, the media was aspirated and cells were treated with accutase enzyme (200 µL/well) for 5 min to dissociate the adhered cells. The protease activity was stopped by adding some medium containing FBS and the cells were collected by centrifugation. After removal of supernatant, cells were washed twice with PBS and incubated with Annexin V-FITC and propidium iodide (PI) for 15 min. The stained cells were detected and quantified using FacsCalibur (Becton Dickinson, San Jose, USA) flow cytometer equipped with 488 nm laser for excitation, and 515 nm and 600 nm band-pass filters for the detection of FITC and PI, respectively. The obtained data were analyzed using CellQuest software (BD Biosciences, US). Annexin V-FITC positive/ PI negative cells were reported as early apoptotic cells.

### Western blotting

For western blotting the transfected cells were washed with pre-chilled PBS and collected by centrifugation (14000 rpm, 5 min). The collected cells were then lysed in RIPA lysis buffer supplemented with 0.1 % protease inhibitor, 0.5 % phosphatase inhibitor and 10 % phenylmethane sulfonyl fluoride (PMSF) (all from Sigma-Aldrich, Germany). The cell suspension was centrifuged at 14000 rpm for 10 min and the total protein in the supernatant was measured using bicinchoninic acid assay (BCA) kit (Thermo Scientific, UK).

Lysates (40 µg protein) were subjected to sodium dodecyl sulfate poly-acrylamide gel electrophoresis (SDS-PAGE) on 8 % poly-acrylamide gel. The separated proteins were then transferred to a poly-vinylidine difluoride membrane (PVDF, Roche Applied Sciences, Germany) by electroblotting. After blocking the membranes with skimmed milk (3 % w/v in TBS) overnight at 4 °C, they were incubated with the primary antibody against PBEF/NAMPT (Rabbit mAb, Cell Signalling Technology, USA) at 1:1000 dilution (overnight, 4°C). The membranes were then incubated for 2 h at room temperature with the secondary antibody (HRP-conjugated anti-rabbit, Cell Signaling Technology, USA) at a dilution of 1:5000. Signals were detected with Clarity Western ECL Substrate (Bio-Rad) followed by exposure to X-ray film for 10 s. The bands intensities were evaluated using ImageJ software and normalized with respect to GAPDH signal intensity as the internal control. 

### NAD assay

The intracellular levels of NAD was measured using NAD Assay Kit (Abcam, UK) according to the instructions provided by the manufacturer. Briefly, transfected cells were lysed and the total protein concentration was measured using BCA protein assay kit as described above. Cell lysates were de-proteinized with perchloric acid to eliminate NADH-consuming enzymes. Total NAD was quantified by enzymatic method and the absorbance of the resulting colored compound was measured at 450 nm. The amount of NAD in each sample was normalized against the total protein of each sample.

### Prediction of miRNA candidates binding to NAMPT mRNA

For the prediction of miRNAs that potentially bind to NAMPT 3′-UTR, the commonly cited prediction programs were used including microrna.org which uses miRanda algorithm for target site prediction (http://www.microrna.org/microrna/home.do), MiRmap (https://mirmap.ezlab.org) (Vejnar and Zdobnov, 2012[24]), miRDB (Wong and Wang, 2014[27]) and TargetScan (http://www.targetscan.org) (Agarwal et al., 2015[2]).

First the NAMPT gene was searched in the above databases and all the microRNAs that potentially bind to their target sites in the 3´-UTR of NAMPT were observed. To choose the microRNAs with higher probability of giving experimentally real and significant results, those that had miRDB score above 60 (according to the recommendation of the database) and miRSVR score below -1.0 (Betel et al., 2010[6]) were selected. Subsequently the expression of the selected microRNAs in normal and tumor tissue of breast was evaluated using miRmine database (http://guanlab.ccmb.med.umich.edu/mirmine/) (Panwar et al., 2017[21]). miRCancer database (http://mircancer.ecu.edu/) (Xie et al., 2013[29], 2015[30]) was also used to investigate the expression of microRNAs in breast cancer as well as other cancer types. 

### Luciferase assay 

The 3′-UTR of NAMPT gene containing the sequence of miR-381 response element (NAMPT-3′-UTR) and its corresponding mutant tandem repeat sequence (NAMPT-MRE-tandem-mut) lacking any complementarity with miR-381, to be used as negative control, were amplified by PCR. All of the primer sets are listed in Supplementary Table 1. They were then cloned downstream of Renilla luciferase in psiCHECK2 vector (Promega, USA), giving rise to NAMPT 3′-UTR-psiCHECK2 and NAMPT-MRE-tandem-mut-psiCHECK2 plasmids. HEK-293T cells were seeded into 12-wells plates and co-transfected with the above plasmids along with miR-381 mimic, miR-381 inhibitor and their NCs using PEI. Meanwhile, transfection with un-cloned psiCHECK2 plasmid either alone or together with miR-381 was performed as control. Cells were also exposed to mock transfection (treatment with PEI transfection reagent alone). After 4 h, DMEM-F12 containing 1 % penicillin-streptomycin and 10 % FBS was added to each well. Subsequently, cells were harvested after 48 h and assayed with a Dual Luciferase assay kit (Promega, USA) according to the manufacturer's instructions. Each value from the Renilla luciferase was normalized to the Firefly luciferase value. 

### Statistical analysis

All experimental data were expressed as means ± standard deviation (SD) of at least three independent experiments. Comparisons between groups were analyzed by one way ANOVA with Tukey's post hoc test using GraphPad Prism software, version 5.01. (USA, San Diego). Statistical significance was determined as *P* ≤ 0.05.

## Results

### miR-381 and NAMPT are inversely expressed in breast cancer cell lines 

**Figure 1:**
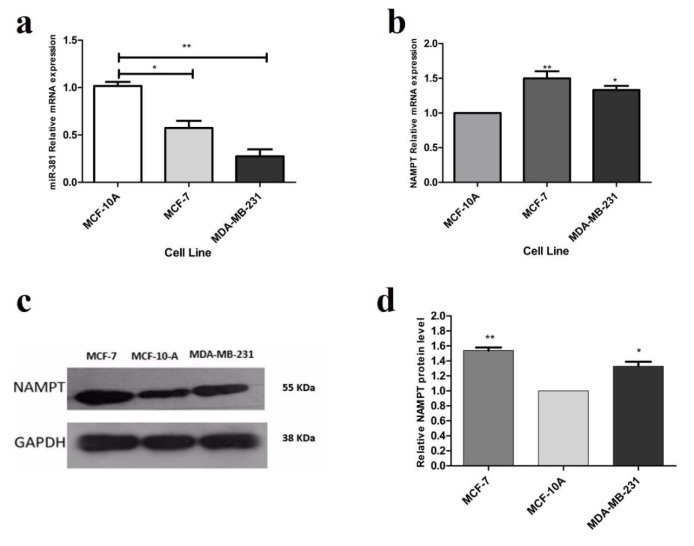
Basal expression of (a) miR-381 and (b) NAMPT in MCF-7 and MDA-MB-231 cell lines, compared with MCF-10A, each vertical bar represents the mean ± SD of triplicate determinations. *P<0.05; **P<0.01. (c) Evaluation of NAMPT basal expression at translational level by immunoblotting in MCF-7 and MDA-MB-231. (d) The basal protein expression levels of NAMPT in MCF-7 and MDA-MB-231 cells compared to MCF-10A cells. Each column represents mean ± SD. *P<0.05; **P<0.01

To obtain insight into the biological role of miR-381 in breast cancer development, its expression was analyzed in human breast cancer (HBC) cell lines and normal breast epithelial cell line (MCF-10A) by RT-PCR. Compared to normal epithelial cell line, miR-381 was significantly under-expressed in MCF-7 (*P*<0.05) and MDA-MB-231 (*P*<0.01) HBC cell lines (Figure 1a(Fig. 1)). This result suggests that down-regulation of miR-381 may be involved in breast cancer development. The comparison between two cell lines revealed that the expression of miR-381 was significantly lower in MDA-MB-231 cells compared to MCF-7 cells. The expression level of NAMPT mRNA was also examined in normal and HBC cell lines using RT-PCR. As shown in Figure 1b(Fig. 1), higher levels of NAMPT mRNA were detected in MCF-7 (*P*<0.01) and MDA-MB-231 (*P*<0.05) compared with MCF-10A. In addition, the blotting results revealed that the basal level of NAMPT protein in MCF-7 and MDA-MB-231 cell lines was 1.53- and 1.28-fold higher than MCF-10A (*P*<0.01 and *P*<0.05), respectively (Figure 1c, d(Fig. 1)).

### miR-381 cellular levels were up-regulated via miRNA mimic transfection

**Figure 2:**
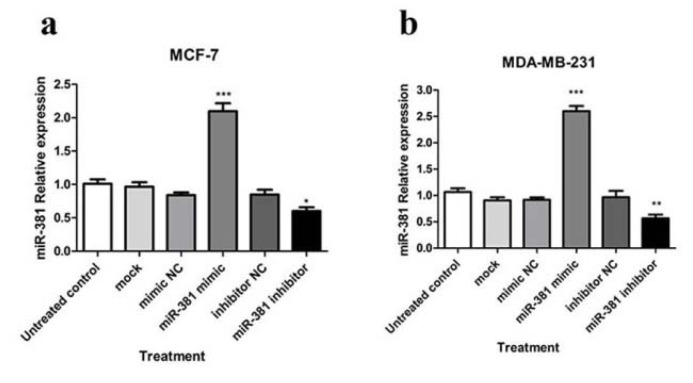
Regulation of miR-381 by miRNA mimic and inhibitor. RT-PCR analysis of miR-381 levels in (a) MCF-7 cells and (b) MDA-MB-231 cells transfected with miR-381 mimic, inhibitor, mock and negative controls (NC). Each column represents mean ± SD of at least three separate experiments. *P<0.05; **P<0.01; ***P<0.001

To understand the mechanism by which miR-381 controls NAMPT expression in HBC cells, transfection with miR-381 mimic and miR-381 inhibitor was conducted. Transfected cells were visualized with fluorescence microscope in order to confirm the successful transfection of FAM-labeled miRNAs (Supplementary Figure 1). The results showed that miRNA mimic significantly increased cellular levels of miR-381 in MCF-7 (*P*<0.001) and MDA-MB-231 (*P*<0.05) cells, while the transfection with miR-381 inhibitor led to a significant decrease in miR-381 levels in HBC cell lines compared to un-treated cells (*P*<0.05 and *P*<0.01, respectively) (Figure 2(Fig. 2)). 

### Relationship between miR-381 and NAMPT at post-transcriptional level 

As described earlier, bioinformatics analysis predicted that 3′-UTR of NAMPT is a target for miR-381. The results of these analyses showed that miR-381 had a 7mer seed site at the position of 359-365 of the NAMPT 3´UTR and aligned with this site with miRSVR score of -1.3 and miRDB score of 69. The results of the search in miRmine database showed that miR-381 is expressed both in the normal and tumor tissue of breast and the results of miRCancer search also indicated that this microRNA is down-regulated not only in breast cancer but also in other types of cancers. The search results in various databases are all included in Supplementary Figures 3-7 (supplementary data). 

Consequently, it was hypothesized that down-regulated miR-381 in cancer cells might be involved in NAMPT up-regulation. To evaluate whether miR-381 would exert an inhibitory effect on NAMPT expression, RT-PCR and Western blotting were performed on HBC cells transfected with miR mimic, miR inhibitor, and their corresponding NCs. 

**Figure 3:**
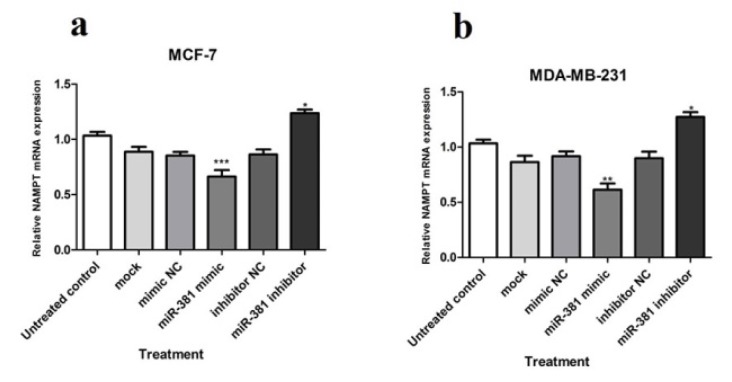
Relative NAMPT mRNA expression in (a) MCF-7 and (b) MDA-MB-231 cells transfected with miR-381 mimic, inhibitor, their negative controls (NC), and mock compared to un-treated cells. Each column represents mean ± SD of at least three separate experiments. *P<0.05; **P<0.01; ***P<0.001

At the mRNA level, *NAMPT* gene displayed a significantly diminished expression in MCF-7 and MDA-MB-231 cell lines (*P*<0.001 and *P*<0.01, respectively) due to miR-381 augmentation by its mimic. Quite the reverse, blocking miR-381 by its corresponding inhibitor caused a significant increase in NAMPT mRNA expression in both MCF-7 and MDA-MB-231 cell lines (both *P*<0.05) (Figure 3a, b(Fig. 3)). 

**Figure 4:**
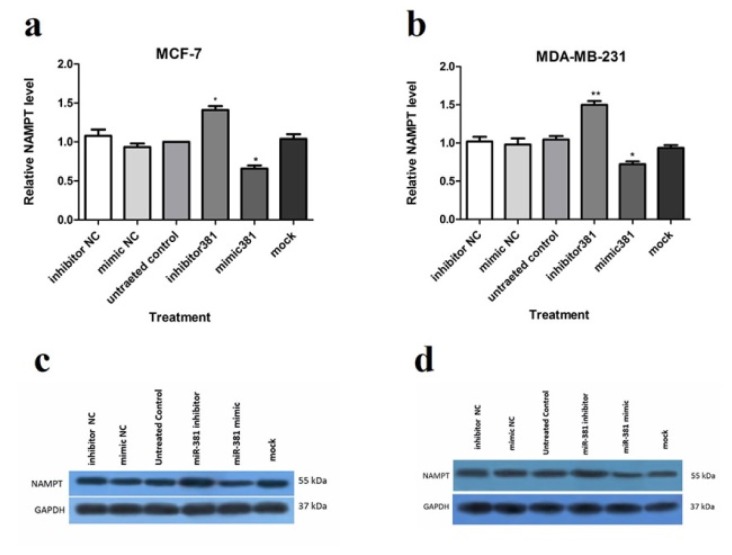
Regulation of NAMPT at mRNA and protein levels via miR-381 mimic transfection. RT-PCR analysis of NAMPT mRNA levels in (a) MCF-7 cells and (b) MDA-MB-231 cells, transfected with miR-381 mimic, inhibitor, their negative controls (NC) and mock compared to un-treated cells. Immunoblot analysis of (c) MCF-7 cells and (d) MDA-MB-231 cells after transfection with miR-381 mimic, inhibitor, their negative controls (NC) and mock compared to un-transfected cells. A representative blot is presented here and the other Western blotting experiments are provided in the supplementary file. Protein bands were quantified using ImageJ software, wherein GAPDH was used as the internal control. The Y-axis represent the density of each band normalized to corresponding GAPDH band relative to control. Each column represents mean ± SD of at least three separate experiments. *P<0.05; **P<0.01; ***P<0.001

Up-regulation of miR-381 significantly suppressed NAMPT protein level in MCF-7 and MDA-MB-231 (both *P*<0.05) compared to the un-treated cells. On the contrary, miR-381 inhibition by its inhibitor led to a significant increase in NAMPT protein level in both MCF-7 (*P*<0.05) and MDA-MB-231 cell lines (*P*<0.01) (Figure 4a, b(Fig. 4) and Supplementary Figure 8). The NCs of mimic and inhibitor did not exert any regulatory effect on NAMPT expression neither at gene nor at the protein levels. 

### miR-381 suppresses NAMPT expression via binding to its 3′-UTR

In order to investigate whether the decreased expression of NAMPT in response to miR-381 was the result of direct binding of miR-381 to the NAMPT 3′-UTR, the luciferase assay was conducted. To this end, the luciferase activity was assessed in the cells co-transfected with miR-381 mimic, inhibitor or their NCs and psiCHECK2 plasmid harboring the wild type or mutated form of miR-381 response element (MRE) in *NAMPT *gene. 

**Figure 5:**
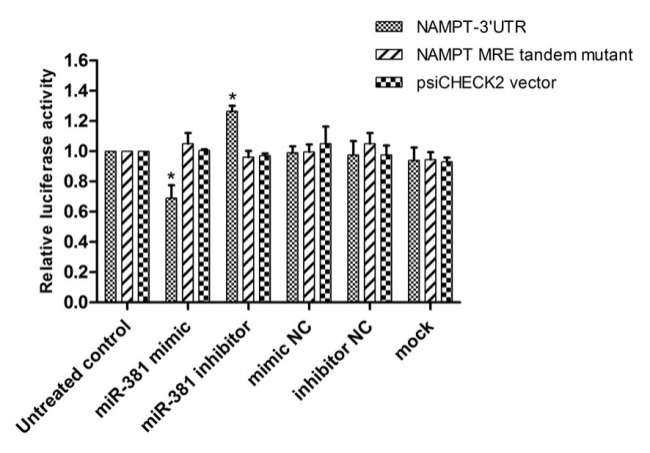
Luciferase reporter assay to determine the binding of miR-381 to NAMPT 3′-UTR. psiCHECK2 plasmids harboring NAMPT 3′-UTR or a mutant tandem repeat of miR-381 response element (MRE-Tandem-Mut) were co-transfected with mimic or inhibitor of miR-381 or their corresponding negative controls (NC) in HEK293T. Renilla luciferase activity was normalized against firefly luciferase as control. Results are shown as mean ± SD of three independent experiments. *P<0.05

The results revealed that miR-381 mimic reduced luciferase activity by approximately 69.0 ± 0.08 % (*P*<0.05). In contrast, down-regulation of miR-381 by its inhibitor, significantly increased luciferase activity (*P*<0.05) (Figure 5(Fig. 5)). The luciferase activity of psiCHECK2 containing the mutant form of MRE was not significantly affected by either miR mimic or inhibitor, ruling out the non-specific bindings (Figure 5(Fig. 5)). Raw data of the luciferase assay is provided in Supplementary Table 2. 

### The effect of miR-381 on NAMPT-induced NAD synthesis 

**Figure 6:**
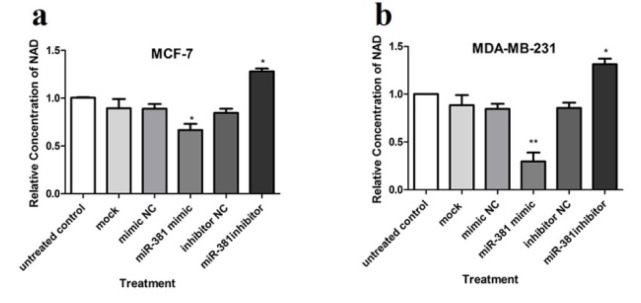
The effect of miR-381 on NAD levels in (a) MCF-7 and (b) MDA-MB-231 cells. Results are presented as mean ± SD of at least three independent experiments. *P<0.05, **P<0.01

NAMPT is a rate-limiting enzyme in NAD biosynthesis pathway (Chen et al., 2013[8]; Hesari et al., 2018[11]). Thus, to verify the effect of NAMPT negative regulation via miR-381 up-regulation, the intracellular level of NAD was assessed. The obtained results showed that transfection with miR-381 mimic led to a significant decrease in NAD levels in both MCF-7 and MDA-MB-231 cell lines (*P*<0.05 and *P*<0.01, respectively) (Figure 6a, b(Fig. 6)). On the other hand, NAD level was significantly augmented after reduction of intracellular miR-381 using its inhibitor in both cell lines (Figure 6a, b(Fig. 6)). 

### The effect of miR-381 on survival and apoptosis of HBC cells

**Figure 7:**
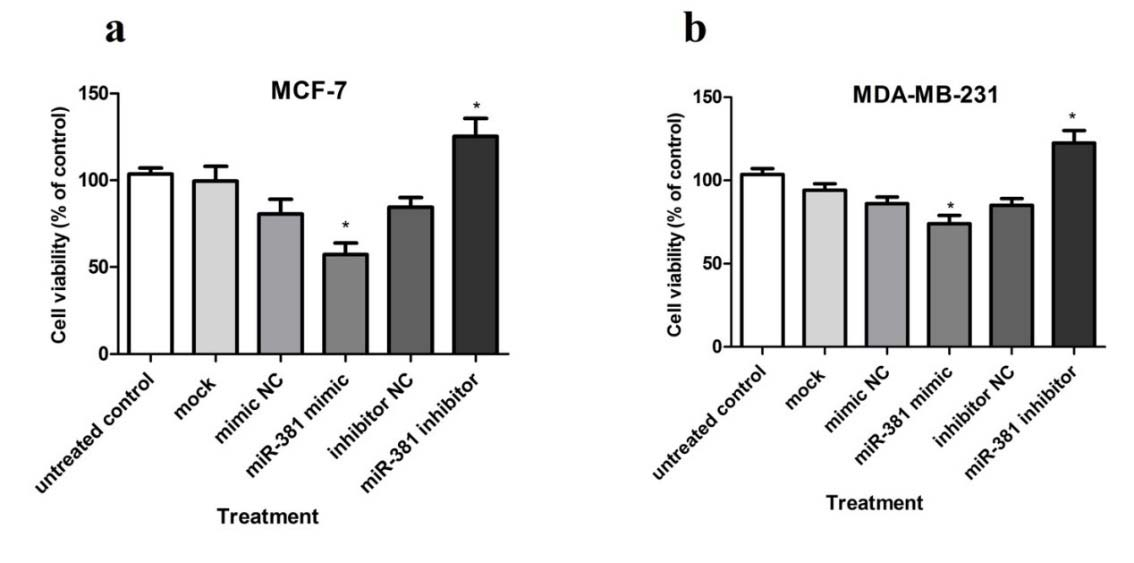
The effect of miR-381 on cell survival in (a) MCF-7 cells and (b) MDA-MB-231 cells transfected with miR-381 mimic, inhibitor and their negative controls (NC) compared with un-treated cells. Data are presented as mean ± SD of three separate experiments. *P<0.05

Taking into account the role of NAMPT in oncogenesis, cancer cell survival and death (Hesari et al., 2018[11]), the effect of miR-381 up-regulation on the viability of HBC cells was evaluated using WST-1 reagent. The results showed that transfection with miR-381 mimic significantly decreased the viability of both MCF-7 and MDA-MB-231 cell lines (P<0.05) compared to un-treated cells (Figure 7a, b(Fig. 7)). Raw data of the viability assay is provided in Supplementary Table 8.

**Figure 8:**
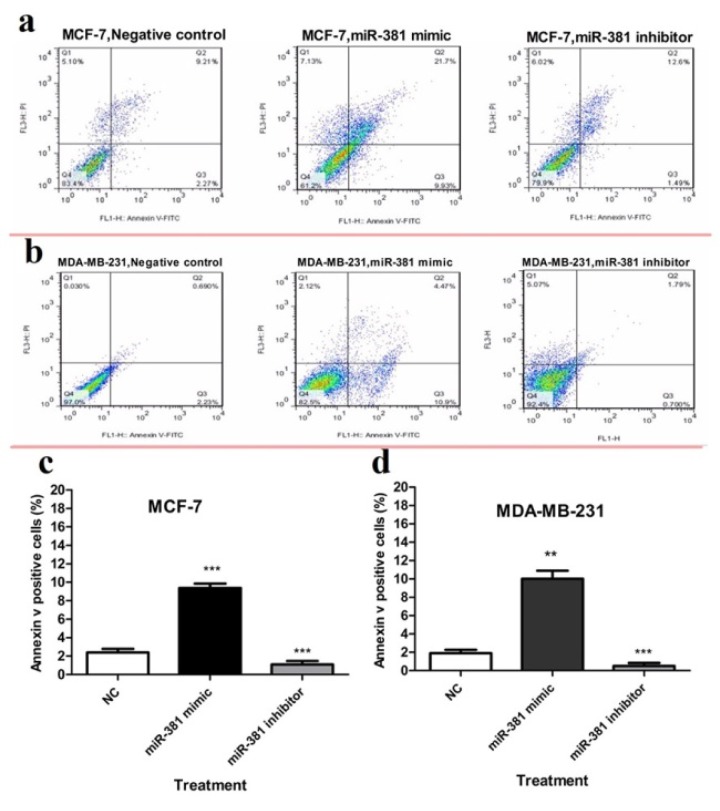
Flow cytometry analysis of annexin-V and propidium iodide (PI) staining of apoptotic cells of (a) MCF-7 and (b) MDA-MB-231 cells transfected with either miR-381 mimic or its inhibitor compared to un-treated control (NC). The average percentage of apoptotic cells in each cell line is shown as mean ± SD. **P<0.01; ***P<0.001

To evaluate apoptosis, cells were subjected to Annexin V and PI double staining followed by flow cytometric analysis. The results demonstrated that miR-381 mimic significantly induced apoptosis in MCF-7 and MDA-MB-231 cell lines (*P*<0.001 and *P*<0.01, respectively), compared to un-transfected cells. On the other hand, treating MCF-7 and MDA-MB-231 cells with miR-381 inhibitor significantly decreased the percentage of early-stage apoptotic cells (*P*<0.001) (Figure 8a, b(Fig. 8)).

## Discussion

In spite of remarkable advances in the diagnosis and treatment of breast cancer, this disease is still the most common malignancy among women all over the world (Al-Hajj et al., 2003[4]). NAD is a vital factor in various cellular processes necessary for cell survival (Sampath et al., 2015[22]). The expression level of NAD-producing enzyme, NAMPT, is dramatically enhanced in cancer patients, particularly those with breast cancer (Dalamaga et al., 2018[9]; Sampath et al., 2015[22]). We had previously shown that inhibition of NAMPT by its chemical inhibitor is an effective approach in reducing breast cancer cell viability and induction of apoptosis (Alaee et al., 2017[3]). Our previous research revealed that NAMPT down-regulation by miR-206 leads to apoptosis in breast cancer cells (Hesari et al., 2018[11]). Here we aimed to investigate the effect of miR-381, which was predicted by bioinformatics tools to target NAMPT, in controlling the growth of cancer cells. 

In the present study, we showed that miR-381 expression was diminished in HBC cell lines compared to normal breast cells and its expression was negatively correlated with NAMPT levels. Consistently, Xue et al. have reported decreased expression of miR-381 in breast cancer cell lines and breast cancer tissue (Xue et al., 2017[32]). The plasma levels of miR-381 is shown to be down-regulated greater than two fold in breast cancer patients (Liu et al., 2013[18]). Several studies have reported low levels of miR-381 in other types of cancers, therefore it can be suggested that miR-381 may function as a tumor suppressor in various cancers. Studies show that the down-regulation of miR-381 in gastric cancer tissues and cell lines is associated with lymph node metastasis, adverse clinic-pathological features, and poor prognosis (Cao et al., 2017[7]). Meanwhile, the miRNA-381 level is found to be significantly reduced in the blood of prostate cancer patients (Cao et al., 2017[7]) and hepatocellular carcinoma cells (HCC) (Zhang et al., 2016[34]). 

The lower expression of miR-381 in MDA-MB-231 cells compared to MCF-7 cells might be the result of different p53 function in these two cell lines. MDA-MB-231 cells carry mutated *TP53* while p53 is functional and active in MCF-7 cells (Negrini et al., 1994[20]). It has been reported that p53 binds to the promoter of some miRNAs including miR-381 and activates their transcription (Liang et al., 2015[17]). Therefore the higher miR-381 expression in MCF-7 cells might be attributed to the inducing effect of p53. In the current study, a negative correlation between miR-381 expressions and NAMPT levels was found, such that NAMPT gene and protein expression levels were decreased in response to miR-381 up-regulation. On the other hand, reducing endogenous cellular miR-381 levels using its inhibitor caused a significant rise in NAMPT levels, further emphasizing the fundamental role of miR-381 in the regulation of NAMPT expression. The results of luciferase reporter assay indicated the direct binding of miR-381 to NAMPT 3′-UTR and rejected the possible off-target and indirect effects of this microRNA. As previously reported by Zhou et al., NAMPT up-regulation is related to tumor size, lymph node metastasis and advanced clinical tumor node metastasis (TNM) stages (Zhou et al., 2018[37]). Manipulation of NAMPT expression is, therefore, a reasonable strategy to overcome breast cancer development and progression. Breast cancer cells especially benefit from NAD depletion by genetic inhibition of NAMPT (Zhang et al., 2009[35]) . 

Our results revealed that negative-regulation of NAMPT by miR-381 induced apoptosis and decreased cell survival in breast cancer cells. NAMPT is strongly associated with cell survival and apoptosis and provides NAD as the substrate for SIRT1 which is one of the major longevity-associated enzymes in the cells (Imai and Guarente, 2016[12]). On the other hand, decreased SIRT1 activity due to NAD depletion causes acetylation and activation of pro-apoptotic factors such as p53, FOXO and poly(ADP-ribose) polymerase 1 (PARP1) (Menssen et al., 2012[19]; Abdolvahabi et al., 2019[1]) Consistently, Zhang et al. reported that miR-26b causes cell death and serves as a tumor suppressor in colorectal cancer cells and concluded that this effect is mediated through the inhibitory effect of this microRNA on NAMPT expression (Chen et al., 2013[8]). 

As discussed above, induction of apoptosis is the hallmark of cancer treatment and attenuated apoptosis causes resistance to anticancer drugs (Wong, 2011[28]). Thus induction of apoptosis by miR-381 can be beneficial for the control of growth of breast cancer cells. 

## Conclusion

The present study suggests that miR-381 negatively regulates NAMPT at the post-transcriptional level by direct binding to its 3′-UTR. Additionally, miR-381 inhibited tumor growth by down-regulation of NAD levels and subsequently promoted apoptosis in breast cancer cells. Taken together, these results indicated that inhibition of NAMPT by miR-381 may have therapeutic potential for the treatment of breast cancer. 

## Notes

Mitra Nourbakhshb and Kazem Mousavizadeh (Pharm.D PhD; Department of Molecular Medicine, Faculty of Advanced Technologies in Medicine, Iran University of Medical Sciences, Tehran, Iran. Cellular and Molecular Research Center, Faculty of Medicine, Iran University of Medical Sciences ,Tehran, Iran; Hemmat Highway 1449614535, Tehran, Iran; Tel: +98-21- 86704720, Fax: +98-21- 88622578, Mobile: +98-9369973054, E-mail: mousavizadeh.k@iums.ac.ir) contributed equally as corresponding authors.

## Acknowledgement

This study was supported by a grant from Iran University of Medical Sciences (grant number 27355).

## Conflict of interest

The authors declare that there are no conflicts of interest.

## Supplementary Material

Supplementary data

## Figures and Tables

**Table 1:**
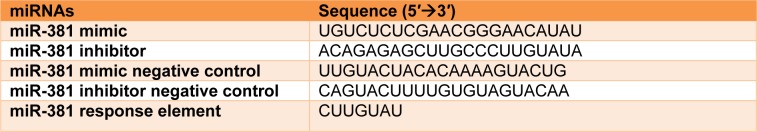
Sequences of miR-381 mimic, miR-381 inhibitor, their corresponding negative controls and miR-381 response element
