# Supplementary material for: Up-regulation of miR-381 inhibits NAD+ salvage pathway and promotes apoptosis in breast cancer cells
Source: EXCLI J. 2019 Aug 27;18:683–96. doi: 10.17179/excli2019-1431 (PMC6785761; doi:10.17179/excli2019-1431)
Supplement: Supplementary data [file EXCLI-18-683-s-001.pdf]

EXCLI Journal 2019;18:683-696 – ISSN 1611-2156
Received: May 12, 2019, accepted: August 14, 2019, published: August 27, 2019

Supplementary data to:

UP-REGULATION OF MIR-381 INHIBITS NAD$^+$ SALVAGE PATHWAY AND PROMOTES APOPTOSIS IN BREAST CANCER CELLS

Zahra Bolandghamat Pour[a], Mitra Nourbakhsh[b,*], Kazem Mousavizadeh[a,d,*], Zahra Madjd[a,e], Seyedeh Sara Ghorbanhosseini[b], Zohreh Abdolvahabi[f] , Zahra Hesari[c,g], Samira Ezzati Mobaser[b]

[a] Department of Molecular Medicine, Faculty of Advanced Technologies in Medicine, Iran University of Medical Sciences, Tehran, Iran
[b] Department of Biochemistry, School of Medicine, Iran University of Medical Sciences, Tehran, Iran
[c] Laboratory Sciences Research Center, Golestan University of Medical Sciences, Gorgan, Iran
[d] Cellular and Molecular Research Center, Faculty of Medicine, Iran University of Medical Sciences, Tehran, Iran
[e] Oncopathology Research Center, Iran University of Medical Sciences, Tehran, Iran
[f] Department of Biochemistry and Genetics, Cellular and Molecular Research Center, Qazvin University of Medical Sciences, Qazvin, Iran
[g] Department of Laboratory Sciences, Faculty of Paramedicine, Golestan University of Medical Sciences, Gorgan, Iran

* **Corresponding authors**: Mitra Nourbakhsh, MSc, PhD, Department of Biochemistry, Faculty of Medicine, Iran University of Medical Sciences, Hemmat Highway 1449614535, Tehran, Iran, Tel: +98 21 86703109, Fax: +98 21 88622742, Mobile: +98 912 2874740, E-mail: nourbakhsh.m@iums.ac.ir
Kazem Mousavizadeh, Pharm.D, PhD, Department of Molecular Medicine, Faculty of Advanced Technologies in Medicine, Iran University of Medical Sciences, Tehran, Iran. Cellular and Molecular Research Center, Faculty of Medicine, Iran University of Medical Sciences ,Tehran, Iran. Hemmat Highway 1449614535, Tehran, Iran,
Tel: +98-21- 86704720, Fax: +98-21- 88622578, Mobile: +98-9369973054,
E-mail: mousavizadeh.k@iums.ac.ir

http://dx.doi.org/10.17179/excli2019-1431

This is an Open Access article distributed under the terms of the Creative Commons Attribution License (http://creativecommons.org/licenses/by/4.0/).

EXCLI Journal 2019;18:683-696 – ISSN 1611-2156
Received: May 12, 2019, accepted: August 14, 2019, published: August 27, 2019

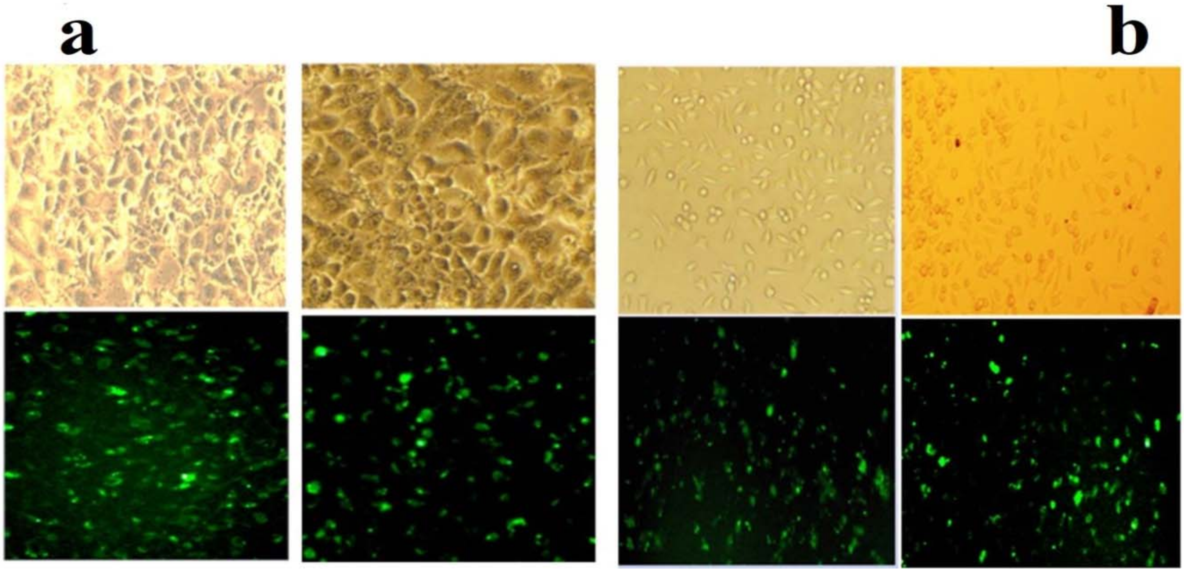

Supplementary Figure 1: Evaluation of transfection efficiency under fluorescent (lower panel) and light (upper panel) microscope by FAM-labeled microRNAs in **a**) MCF-7 and **b**) MDA-MB-231 cells

MicroRNA and Target Gene Description:

**miRNA Name**	hsa-miR-381-3p	**miRNA Sequence**	UAUACAAGGGCAAGCUCUCUGU
**Previous Name**	hsa-miR-381		
**Target Score**	69	**Seed Location**	359
**NCBI Gene ID**	10135	**GenBank Accession**	NM_005746
**Gene Symbol**	NAMPT	**3' UTR Length**	2809
**Gene Description**	nicotinamide phosphoribosyltransferase		

3' UTR Sequence

```
  1 gctttatgac tgggtgtgtg ttgtgtgtat gtaatacata atgtttattg tacagatgtg
 61 tggggtttgt gttttatgat acattacagc caaattattt gttggtttat ggacatactg
121 ccctttcatt ttttttcttt tccagtgttt aggtgatctc aaattaggaa atgcatttaa
181 ccatgtaaaa gatgagtgct aaagtaagct ttttagggcc ctttgccaat aggtagtcat
241 tcaatctggt attgatcttt tcacaaataa cagaactgag aaacttttat atataactga
301 tgatcacata aaacagattt gcataaaatt accatgattg ctttatgttt atatttaact
361 tgtattttg tacaaacaag attgtgtaag atatatttga agtttcagtg atttaacagt
421 ctttccaact tttcatgatt tttatgagca cagactttca agaaaatact tgaaaataaa
481 ttacattgcc ttttgtccat taatcagcaa ataaaacatg gccttaacaa agttgtttgt
541 gttattgtac aatttgaaaa ttatgtcggg acataccta tagaattact aaccttactg
601 ccccttgtag aatatgtatt aatcattcta cattaaagaa aataatggtt cttactggaa
661 tgtctaggca ctgtacagtt attatatatc ttggttgttg tattgtacca gtgaaatgcc
721 aaatttgaaa ggcctgtact gcaattttat atgtcagaga ttgcctgtgg ctctaatatg
781 cacctcaaga ttttaaggag ataatgtttt tagagagaat ttctgcttcc actatagaat
```

Supplementary Figure 2: The 3´-UTR of NAMPT as a target of NAMPT and miR-381 response element in the 3´UTR of NAMPT as found in miRDB database (http://mirdb.org/cgi-bin/target_detail.cgi?targetID=1808567)

EXCLI Journal 2019;18:683-696 – ISSN 1611-2156
Received: May 12, 2019, accepted: August 14, 2019, published: August 27, 2019

a.

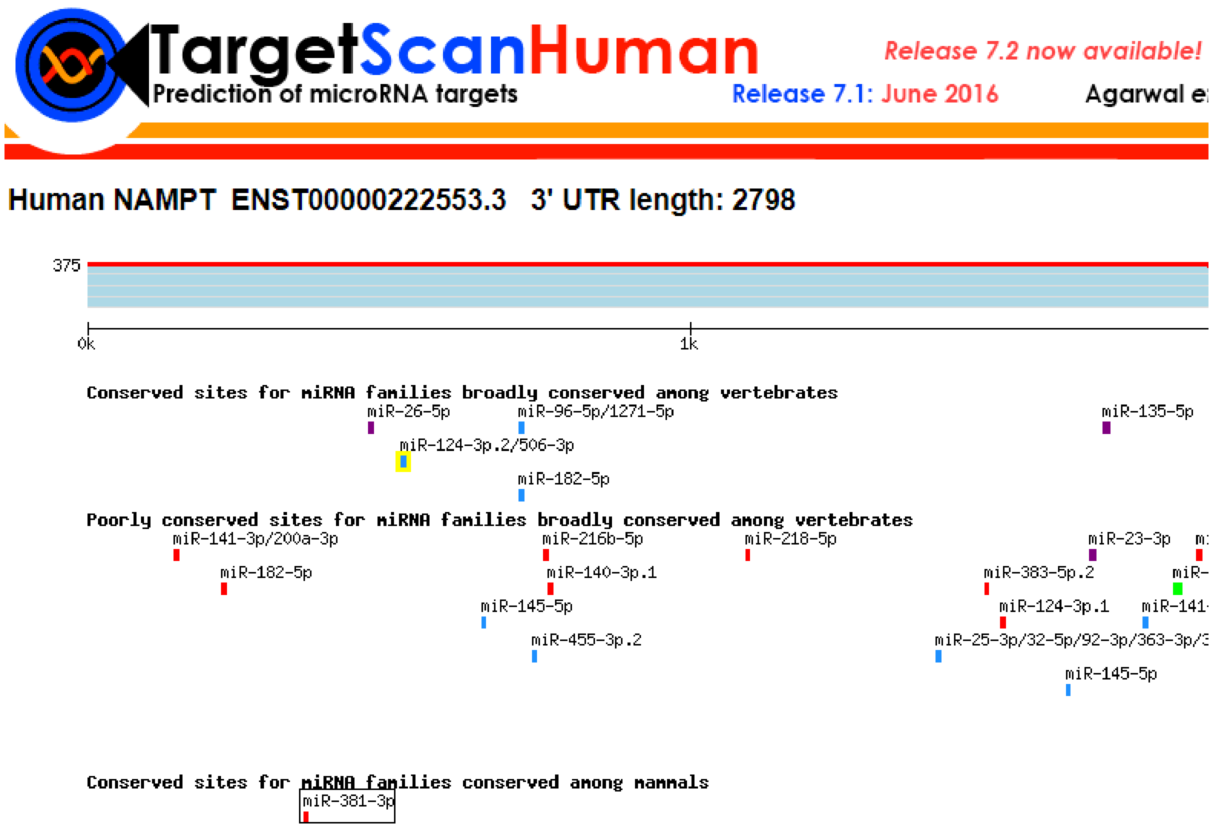

b.

	Predicted consequential pairing of target region (top) and miRNA (bottom)	Site type	Context++ score	Context++ score percentile	Weighted context++ score	Conserved branch length
Position 359-365 of NAMPT 3' UTR<br>hsa-miR-381-3p	5' ...UAUGUUUAUAUUUAACUUGUAUU...<br>||||||| 3' UGUCUCUCGAACGGGAACAUAU	7mer-m8	-0.16	97	-0.16	3.038

Supplementary Figure 3:

a) Targeting of NAMPT 3´UTR by miR381-3p as presented by Target Scan database (http://www.targetscan.org/cgi-bin/targetscan/vert_71/view_gene.cgi?rs=ENST00000222553.3&taxid=9606&members=miR-381-3p&showcnc=1&shownc=1&subset=1). miR-381 is indicated in frame.

b) Pairing of miR-381-3p with its seed sequence and its position in the NAMPT 3'UTR presented by the same database

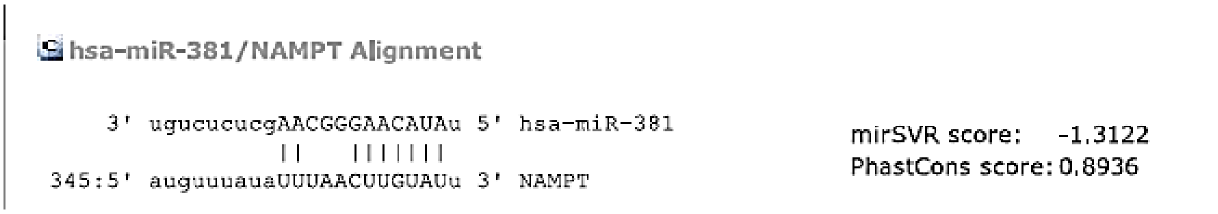

Supplementary Figure 4: Alignment of miR-381 with NAMPT and its mirSVR score as presented by www.microRNA.org (last update 2010.11.01) (http://www.microrna.org/microrna/home.do)

EXCLI Journal 2019;18:683-696 – ISSN 1611-2156
Received: May 12, 2019, accepted: August 14, 2019, published: August 27, 2019

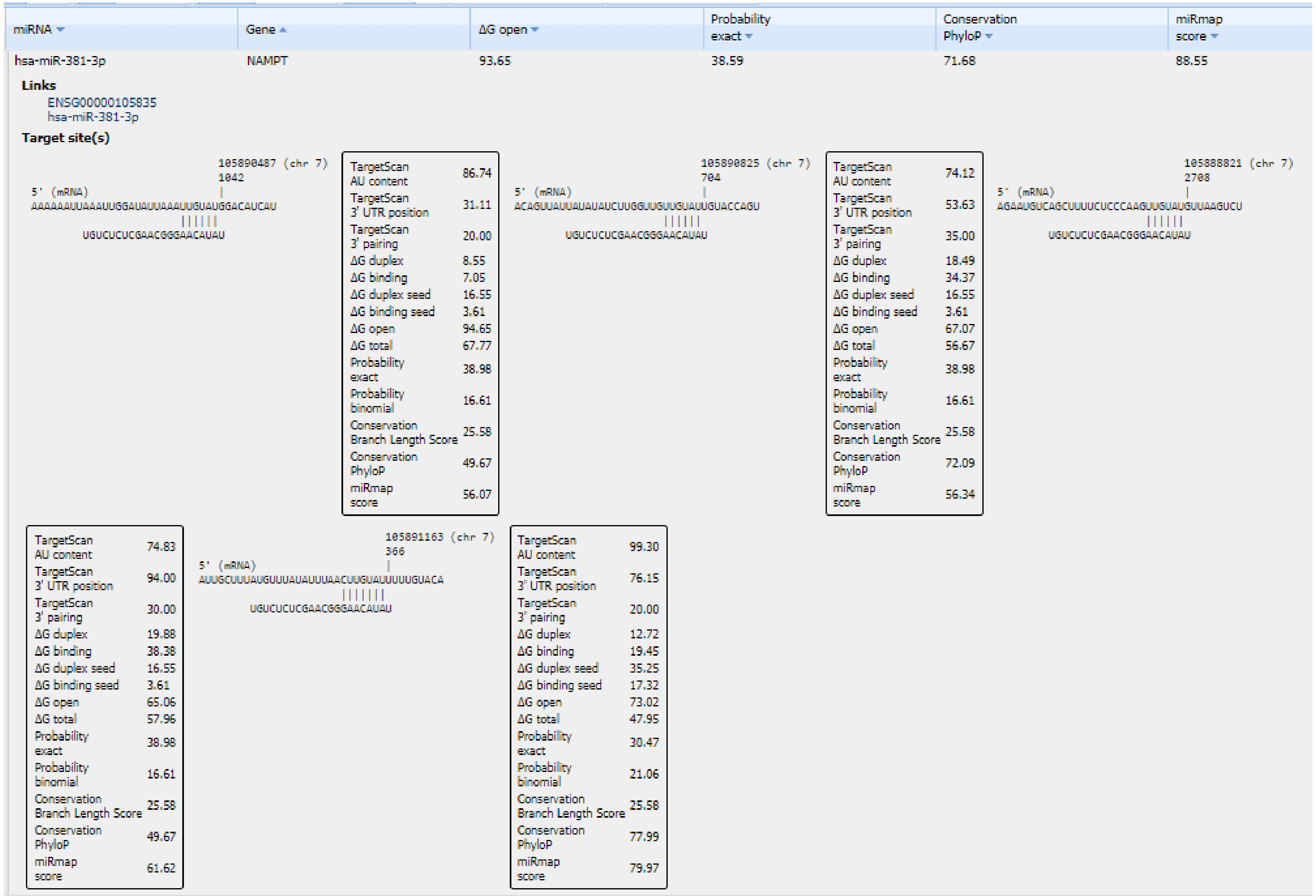

Supplementary Figure 5: Alignment of miR-381-3p with the 3´-UTR of NAMPT and its properties as presented by miRmap (https://mirmap.ezlab.org/app/)

Expression values (RPM) of hsa-miR-381-3p in breast

Tissue	Description	Disease	Sex	PubMed ID
Breast	Serum from healthy woman control	Normal		24904649
Breast	Tumor serum	Breast cancer		24904649
Breast	Tumor tissue	Breast cancer		24904649
Breast	Normal tissue	Normal		24904649

Supplementary Figure 6: Expression of miR-381-3p in normal and tumor tissue of breast (http://guanlab.ccmb.med.umich.edu/mirmine/single.php?mirna=hsa-miR-381-3p&tissue=breast&cline=)

Search result for miRNA = 'hsa-miR-381' Or cancer = '' Total: 13 relations found.

mirId	Family/Cluster	Cancer	Profile	PubMed Article
hsa-mir-381		breast cancer	down	miR-381 suppresses C/EBP?-dependent Cx43 expression in breast cancer cells.
hsa-mir-381		breast cancer	down	Analysis of miR-205 and miR-155 expression in the blood of breast cancer patients.
hsa-mir-381		colon cancer	down	Down-regulation of MicroRNA-381 promotes cell proliferation and invasion in colon cancer through up-regulation of LRH-1.
hsa-mir-381		colon cancer	down	[Expression and proliferative regulation of miR-204 related to mitochondrial transcription factor A in colon cancer].
hsa-mir-381		colorectal cancer	down	MiR-381 functions as a tumor suppressor in colorectal cancer by targeting Twist1.
hsa-mir-381		endometrial cancer	down	MicroRNA-381 inhibits cell proliferation and invasion in endometrial carcinoma by targeting the IGF-1R.
hsa-mir-381		esophageal squamous cell carcinoma	down	MicroRNA-381 enhances radiosensitivity in esophageal squamous cell carcinoma by targeting X-linked inhibitor of apoptosis protein.
hsa-mir-381		gastric cancer	down	MicroRNA-381 inhibits the metastasis of gastric cancer by targeting TMEM16A expression.
hsa-mir-381		gastric cancer	down	MiR-381 inhibits migration and invasion in human gastric carcinoma through downregulatedting SOX4.
hsa-mir-381		hepatocellular carcinoma	down	MicroRNA-381 suppresses cell growth and invasion by targeting the liver receptor homolog-1 in hepatocellular carcinoma.
hsa-mir-381		lung adenocarcinoma	down	MicroRNA-381 represses ID1 and is deregulated in lung adenocarcinoma.
hsa-mir-381		non-small cell lung cancer	down	microRNA-381 suppresses the growth and increases cisplatin sensitivity in non-small cell lung cancer cells through inhibition of nuclear factor-?B signaling.
hsa-mir-381		osteosarcoma	down	MicroRNA-381 suppresses the proliferation of osteosarcoma cells through LRH-1/Wnt/?-catenin signaling pathway.

Supplementary Figure 7: The expression status of miR-381 in breast and other cancer types (http://mircancer.ecu.edu/search.jsp?mirId=hsa-miR-381&logic=&condition=Or&cancerName=&buttonSearch=)

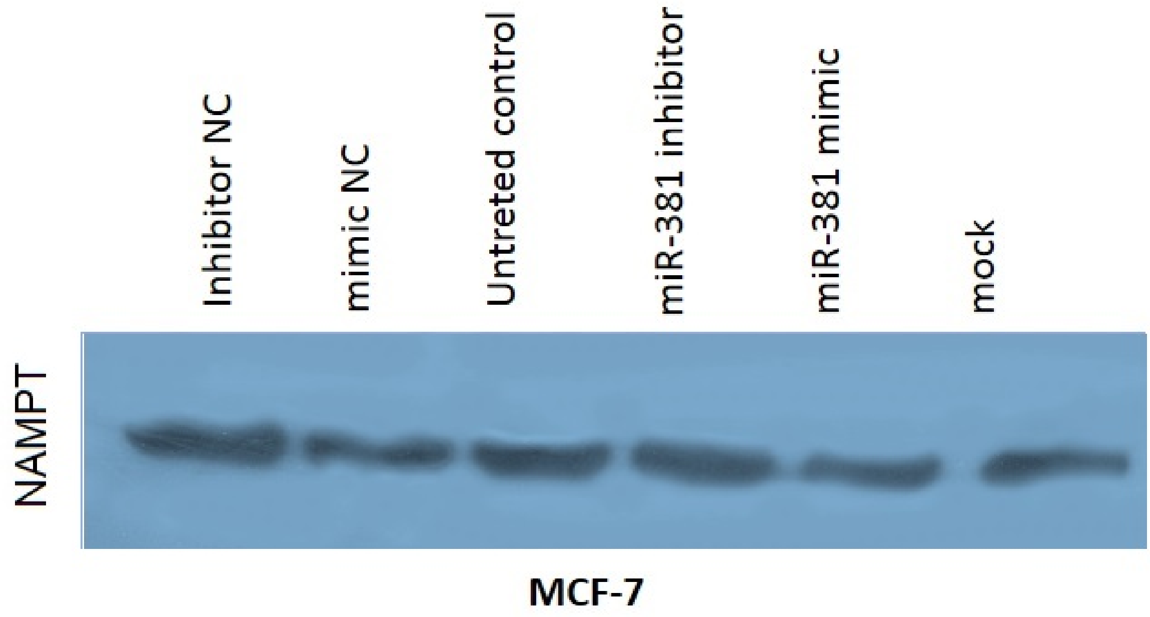

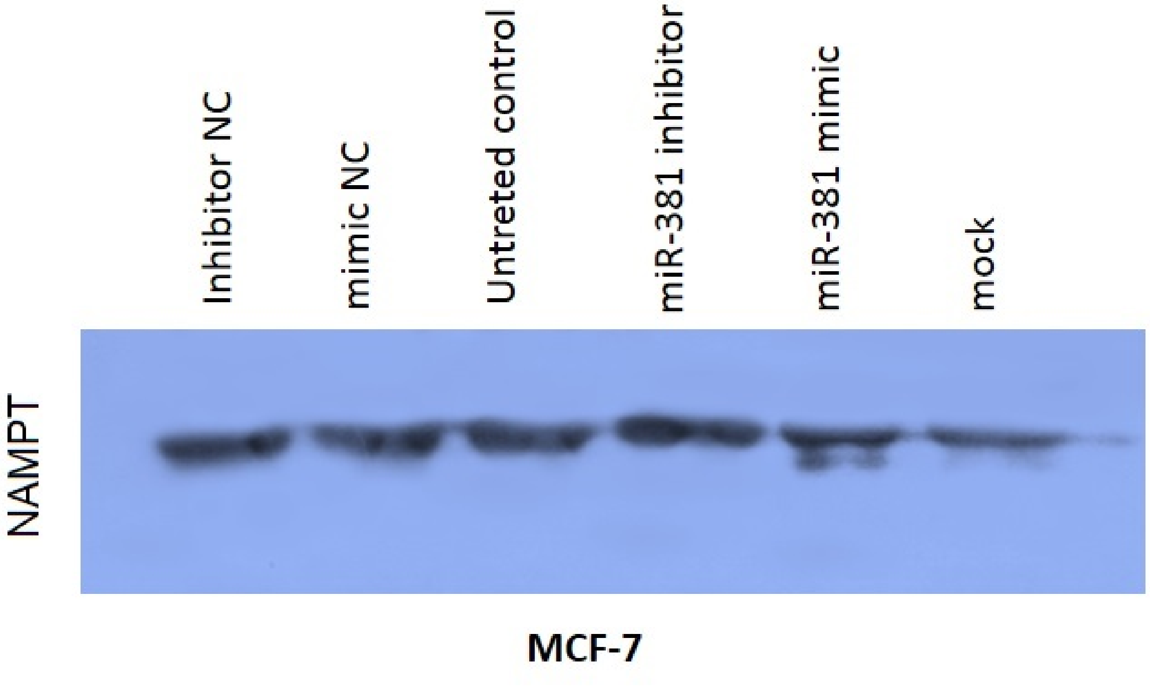

Supplementary Figure 8: Individual images of each Western blot experiment. The cell line in which the experiment was performed is stated under each blot.

EXCLI Journal 2019;18:683-696 – ISSN 1611-2156
Received: May 12, 2019, accepted: August 14, 2019, published: August 27, 2019

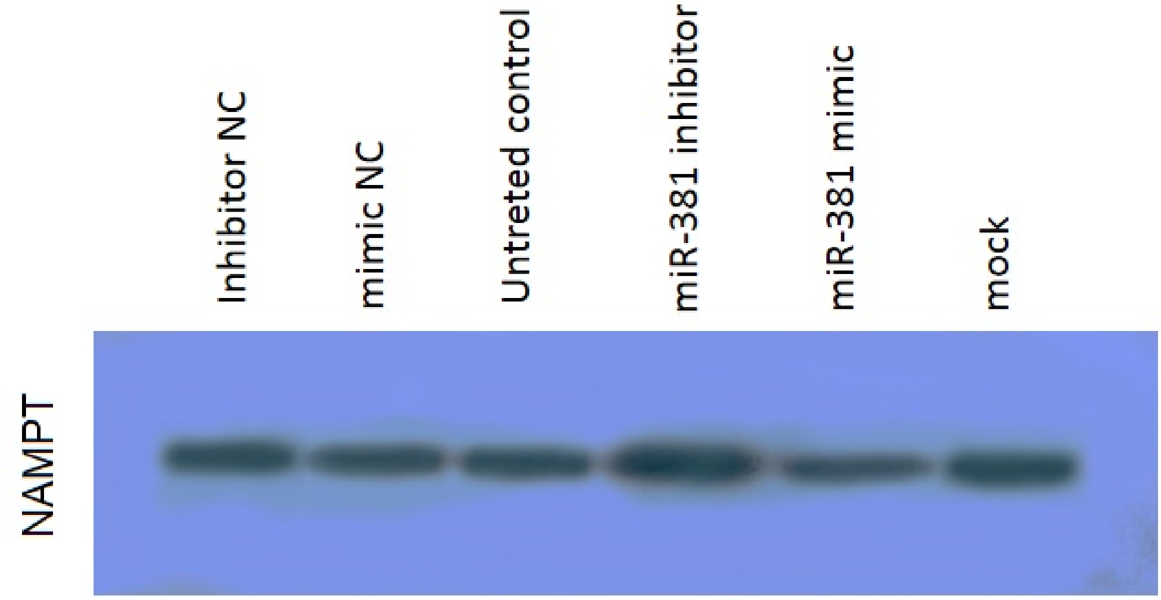

MCF-7

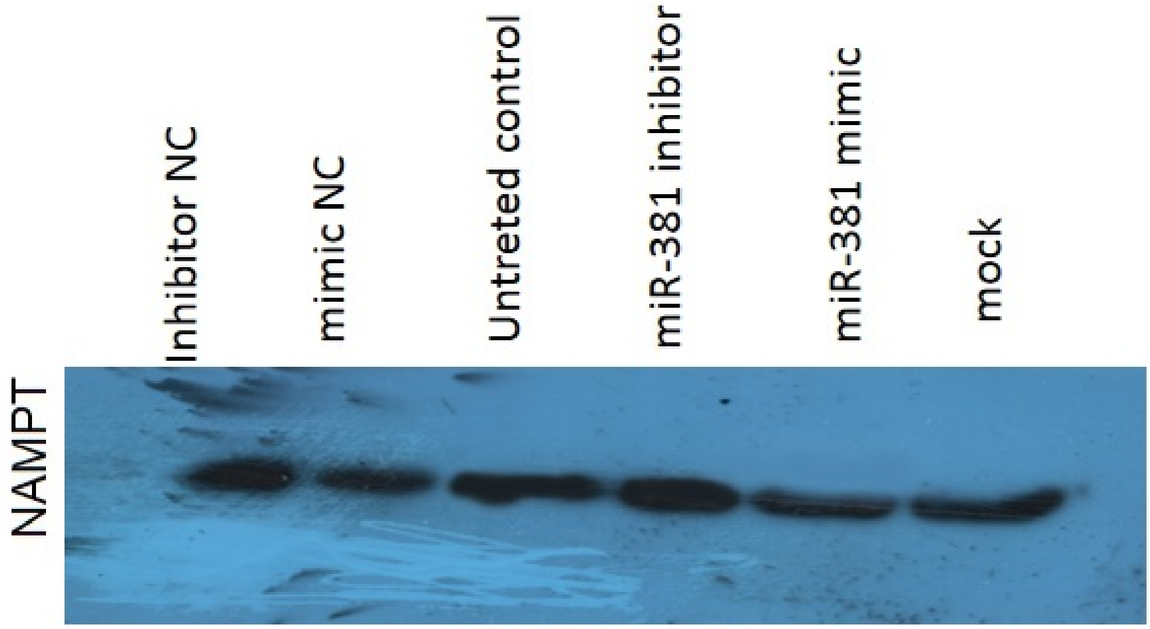

MDA-MB-231

Supplementary Figure 8 (cont.): Individual images of each Western blot experiment. The cell line in which the experiment was performed is stated under each blot.

EXCLI Journal 2019;18:683-696 – ISSN 1611-2156
Received: May 12, 2019, accepted: August 14, 2019, published: August 27, 2019

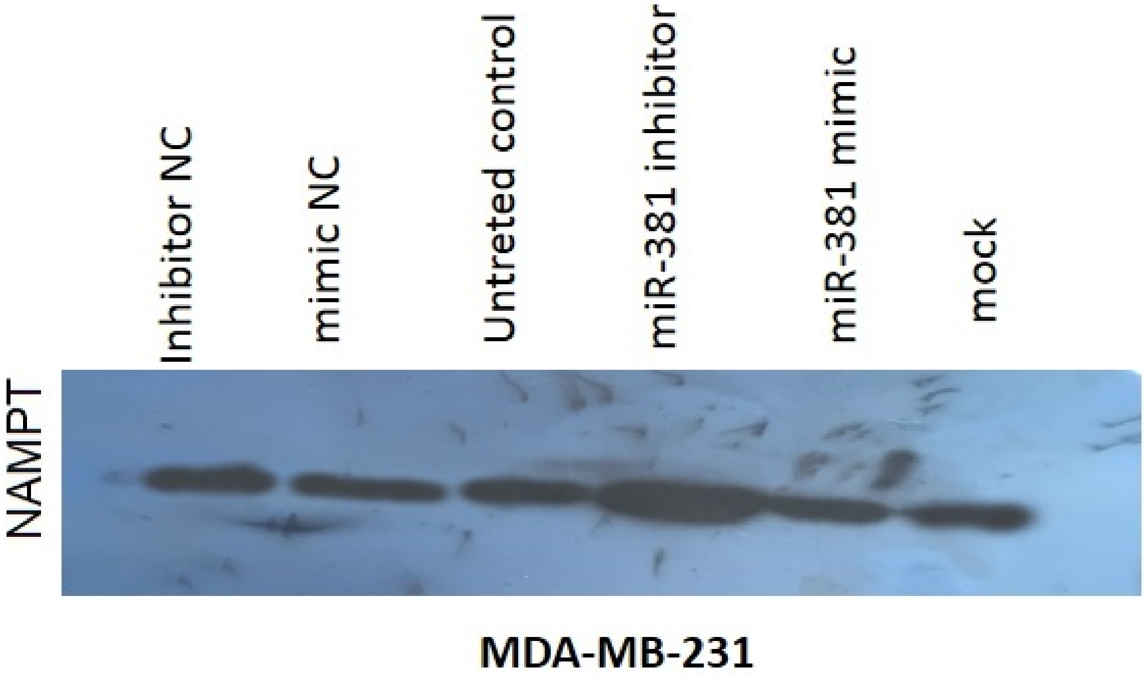

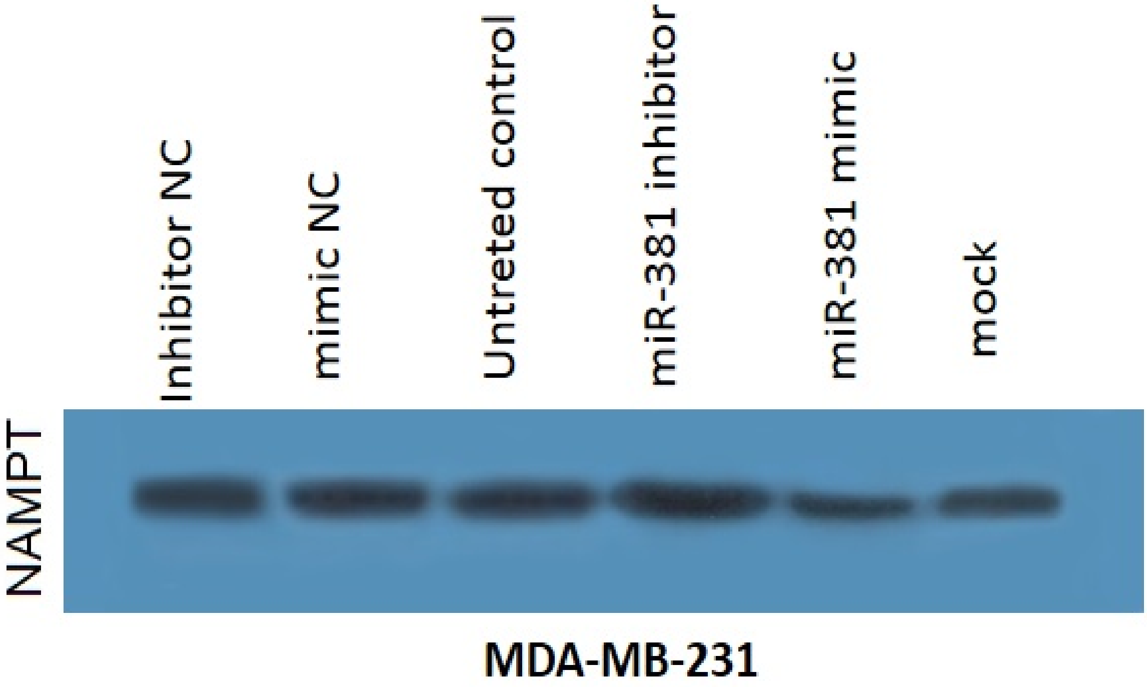

Supplementary Figure 8 (cont.): Individual images of each Western blot experiment. The cell line in which the experiment was performed is stated under each blot.

EXCLI Journal 2019;18:683-696 – ISSN 1611-2156
Received: May 12, 2019, accepted: August 14, 2019, published: August 27, 2019

Supplementary Table 1: Sequences of primers used for measuring miRNA and NAMPT expression, synthesis of NAMPT 3′-UTR and NAMPT MRE tandem mutant

Template	*Primer*	*Sequence (5′→3′)*
miRNA		
miR-381	Forward	AGTATACAAGGGCAAGCTCTCTGT
	Universal Reverse	GCGAGCACAGAATTAATACGACTC
U6-snRNA	Forward	CTCGCTTCGGCAGCACA
(Internal Control)	Reverse	AACGCTTCACGAATTTGCGT
Reverse Transcription		GCGAGCACAGAATTAATACGACTCAC-TATAGGTTTTTTTTTTTTAG
Genes		
NAMPT	Forward	GGTTCTTGGTGGAGGTTTGCTAC
	Reverse	GAAGACGTTAATCCCAAGGCC
GAPDH	Forward	GGGAAGGTGAAGGTCGGAGT
(Internal Control)	Reverse	TCCACTTTACCAGAGTTAAAAGCAG
NAMPT-3´-UTR	Forward	CCGCTCGAGCGGGTACAGATGTGTGGGGTTTGTG
	Reverse	AAATATGCGGCCGCGCTGACATTCTCCACTGAATGGG
NAMPT MRE Tandem Mutant	Forward	CCGCTCGAGCGCTTTCACTTTCACTTCTCTTTTTCAC-TTTCACTTCTCTTCTCTCTTCCGTCC
	Reverse	ATAAGAATGCGGCCGCAGGGAGGGGAAAATGAGGAC-GGAAGAGAG

Supplementary Table 2: Raw data of luciferase assay analysis

	NAMPT-3'UTR			NAMPT MRE tandem mutant			psiCHECK2 vector		
	Mean	SD	N	Mean	SD	N	Mean	SD	N
Untreated control	1	0	3	1	0	3	1	0	3
miR-381 mimic	0.693267	0.0814	3	1.033333	0.057735	3	1.02	0.026457	3
miR-381 inhibitor	1.276667	0.032145	3	0.955	0.031225	3	0.972333	0.010786	3
mimic NC	0.993333	0.030551	3	0.986667	0.037859	3	1.036667	0.083267	3
inhibitor NC	0.999333	0.077468	3	1.033667	0.057449	3	0.983333	0.047258	3
mock	0.960333	0.069573	3	0.953333	0.037859	3	0.948	0.037041	3

Received: May 12, 2019, accepted: August 14, 2019, published: August 27, 2019

Supplementary Table 3: Raw data of real-time PCR analysis. miR-381 expression in MCF-7, MDA-MB-231 and MCF-10 cells and their comparison

	U6	miR-381	ΔCt Value	ΔΔCt	Expression Fold Change (2^-ΔΔCt)
	21.97	33.49	11.52	0.2934	0.815976778
	21.78	33.03	11.25	0.0234	0.983911186
MCF10-A	20.66	31.78	11.12	-0.1066	1.076687814
	24.12	36.52	12.4	1.1734	0.443375206
	24.97	36.81	11.84	0.6134	0.653654418
MCF-7	25.65	37.74	12.09	0.8634	0.549655657
	23.51	36.22	12.71	1.4834	0.357644956
MDA-MB-231	22.47	35.57	13.1	1.8734	0.272929454
	24.53	37.98	13.45	2.2234	0.214136109

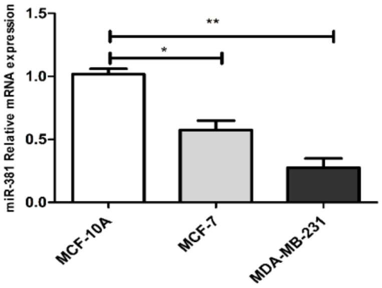

Supplementary Table 4: Raw data showing miR-381 relative expression after transfection of MCF-7 cells with the mimic or inhibitor of miR-381 or their corresponding negative control.

	U6	miR-381	ΔCt Value	ΔΔCt	Expression Fold Change (2^-ΔΔCt)
	21.48	32.54	11.06	-0.123	1.088997015
untreated control	20.19	31.44	11.25	0.067	0.954621014
	21.83	32.97	11.14	-0.043	1.030253954
	22.71	33.7	10.99	-0.193	1.143138335
	24.02	35.21	11.19	0.007	0.995159722
mock	23.65	34.94	11.29	0.107	0.928516852
	24.81	36.22	11.41	0.227	0.854409741
	23.97	35.37	11.4	0.217	0.860352631
mimic NC	24.03	35.28	11.25	0.067	0.954621014
	19.29	29.25	9.96	-1.223	2.334316204
miR-381 mimic	18.75	28.86	10.11	-1.073	2.103803558
	20.54	30.81	10.27	-0.913	1.882956929
	22.19	33.88	11.69	0.507	0.703684188
inhibitor NC	23.54	34.87	11.33	0.147	0.90312651
	24.01	35.21	11.2	0.017	0.988285652
	25.57	37.45	11.88	0.697	0.616853585
miR-381 inhibitor	25.96	37.97	12.01	0.827	0.563700206
	24.53	36.15	11.62	0.437	0.738669032

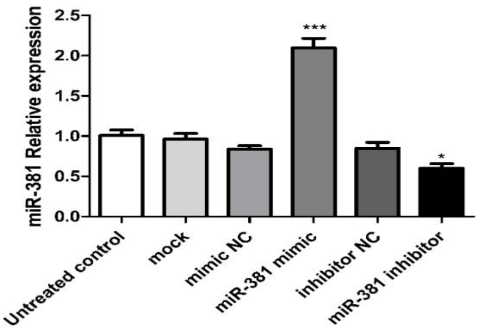

EXCLI Journal 2019;18:683-696 – ISSN 1611-2156
Received: May 12, 2019, accepted: August 14, 2019, published: August 27, 2019

Supplementary Table 5: Raw data showing miR-381 relative expression after transfection of MDA-MB-231 cells with the mimic or inhibitor of miR-381 or their corresponding negative controls (NC)

MDA-MB-231	GAPDH	NAMPT	ΔCt Value	ΔΔCt	Expression Fold Change (2^-ΔΔCt)
untreated control	12.88	23.62	10.74	-0.21	1.156688184
	11.89	22.58	10.69	-0.26	1.197478705
	12.28	23.13	10.85	-0.1	1.071773463
mock	13.01	24.22	11.21	0.26	0.835087919
	12.37	23.65	11.28	0.33	0.795536484
	12.18	23.18	11	0.05	0.965936329
mimic NC	13.37	24.89	11.52	0.57	0.673616788
	13.18	24.28	11.1	0.15	0.901250463
	14.88	26.11	11.23	0.28	0.823591017
miR-381 mimic	14.48	25.98	11.5	0.55	0.683020128
	14.18	26.09	11.91	0.96	0.514056913
	15.09	26.71	11.62	0.67	0.628506687
inhibitor NC	12.76	23.87	11.11	0.16	0.895025071
	13.01	24.11	11.1	0.15	0.901250463
	13.17	24.11	10.94	-0.01	1.00695555
miR-381 inhibitor	11.25	21.76	10.51	-0.44	1.356604327
	10.67	21.25	10.58	-0.37	1.292352831
	10.68	21.25	10.57	-0.38	1.301341855

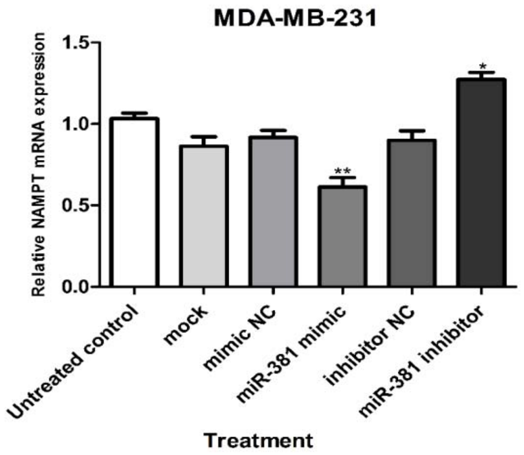

Supplementary Table 6: Raw data showing relative expression of NAMPT mRNA in MCF-7 cells after transfection with miR-381 mimic, inhibitor or NCs

MCF-7	GAPDH	NAMPT	ΔCt Value	ΔΔCt	Expression Fold Change (2^-ΔΔCt)
untreated control	16.87	27.87	11	-0.11	1.079228237
	16.09	27.21	11.12	0.01	0.993092495
	17.08	28.15	11.07	-0.04	1.028113827
mock	19.11	30.35	11.24	0.13	0.91383145
	18.81	29.97	11.16	0.05	0.965936329
	19.15	30.58	11.43	0.32	0.801069878
mimic NC	16.88	28.04	11.16	0.05	0.965936329
	17.87	29.01	11.14	0.03	0.979420298
	17.35	28.65	11.3	0.19	0.876605721
miR-381 mimic	19.32	30.87	11.55	0.44	0.737134609
	18.25	29.89	11.64	0.53	0.692554734
	18.77	30.46	11.69	0.58	0.668963777
inhibitor NC	16.57	27.82	11.25	0.14	0.907519155
	16.44	27.81	11.37	0.26	0.835087919
	17.64	28.96	11.32	0.21	0.864537231
miR-381 inhibitor	16.88	27.65	10.77	-0.34	1.265756594
	17.25	28.01	10.76	-0.35	1.274560627
	15.48	26.15	10.67	-0.44	1.356604327

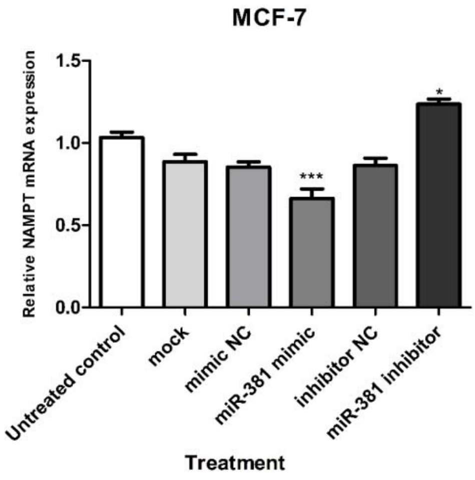

Received: May 12, 2019, accepted: August 14, 2019, published: August 27, 2019

Supplementary Table 7: Raw data showing relative expression of NAMPT mRNA in MDA-MB-231 cells after transfection with miR-381 mimic, inhibitor or NCs

	U6	miR-381	ΔCt Value	ΔΔCt	Expression Fold Change (2^-ΔΔCt)
untreated control	**25.96**	36.97	11.01	-0.093	1.066585781
	**26.21**	37.37	11.16	0.057	0.961260928
	**21.83**	32.97	11.14	-0.043	1.030253954
mock	**24.81**	35.97	11.16	0.057	0.961260928
	**23.02**	34.21	11.19	0.087	0.941478465
	**25.87**	37.24	11.37	0.187	0.878430468
mimic NC	**28.18**	39.25	11.07	-0.033	1.02313747
	**27.19**	38.36	11.17	0.067	0.954621014
	**26.82**	38.18	11.36	0.257	0.836826243
miR-381 mimic	**16.58**	26.36	9.78	-1.323	2.50185816
	**17.41**	27.01	9.6	-1.503	2.834314793
	**16.85**	26.58	9.73	-1.373	2.590085998
inhibitor NC	**25.36**	36.74	11.38	0.277	0.825305409
	**24.67**	35.87	11.2	0.097	0.934975198
	**25.39**	36.28	10.89	-0.213	1.159095952
miR-381 inhibitor	**24.18**	36.15	11.97	0.867	0.548285794
	**23.68**	35.67	11.99	0.887	0.540737382
	**24.39**	36.08	11.69	0.507	0.703684188

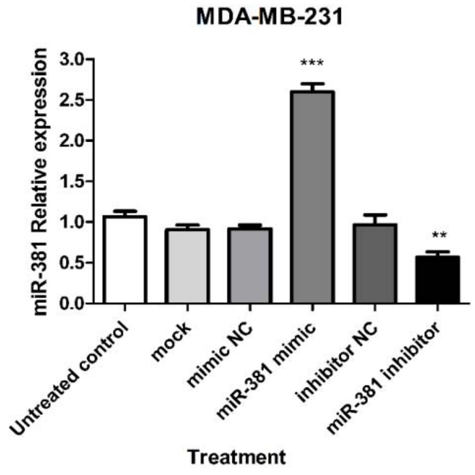

EXCLI Journal 2019;18:683-696 – ISSN 1611-2156
Received: May 12, 2019, accepted: August 14, 2019, published: August 27, 2019

Supplementary Table 8: Raw data of viability assay analysis in MCF-7 and MDA-MB-231 cells respectively

	MCF-7							
0.416	0.504	0.46	0.46	0.36	0.854295	85.42952	inhibitor NC	
0.546	0.542	0.561	0.549667	0.549667	1.304383	130.4383	miR-381 inhibitor	
0.541	0.537	0.524	0.534	0.434	1.0299	102.99	mock	
0.435	0.551	0.548	0.511333	0.411333	0.976111	97.61114	mimin NC	
0.435	0.418	0.324	0.392333	0.292333	0.693719	69.37193	miR-381 mimic	
	mda-mb-231							
0.236	0.254	0.233	0.241	0.141	0.79661	79.66102	inhibitor NC	
0.212	0.315	0.268	0.265	0.165	0.932203	93.22034	mimin NC	
0.252	0.235	0.253	0.246667	0.146667	0.828625	82.86252	miR-381 mimic	
0.277	0.247	0.253	0.259	0.159	0.898305	89.83051	mock	
0.332	0.287	0.367	0.328667	0.228667	1.291902	129.1902	miR-381 inhibitor	
